# *catena*-Poly[copper(II)-bis(μ-2,4-dichloro-6-formyl­phenolato)-κ^3^ *O*,*O*′:*Cl* ^4^;κ^3^ *Cl* ^4^:*O*,*O*′]

**DOI:** 10.1107/S1600536808020424

**Published:** 2008-07-09

**Authors:** Ying Fan, Wei You, Jian-Lan Liu, Hui-Fen Qian, Wei Huang

**Affiliations:** a College of Sciences, Nanjing University of Technology, Nanjing 210009, People’s Republic of China; b State Key Laboratory of Coordination Chemistry, Coordination Chemistry Institute, School of Chemistry and Chemical Engineering, Nanjing University, Nanjing 210093, People’s Republic of China

## Abstract

In the title compound, [Cu(C_7_H_3_Cl_2_O_2_)_2_]_*n*_, the Cu^II^ atom lies on a centre of inversion and adopts a [4+2] coordination mode, with two long axial Cu—Cl coordinative bonds complementing four Cu—O bonds from two 2,4-dichloro-6-formyl­phenolate ligands in a distorted square plane. π–π stacking inter­actions are also formed between neighbouring aromatic rings, with a centroid–centroid separation of 3.624 (2) Å.

## Related literature

For related compounds, see: Duan *et al.* (2007 ▶); Fan, You, Liu *et al.* (2008 ▶); Fan, You, Qian *et al.* (2008 ▶); Harkat *et al.* (2008 ▶); Sun & Gao (2005 ▶); Zhang *et al.* (2006 ▶).
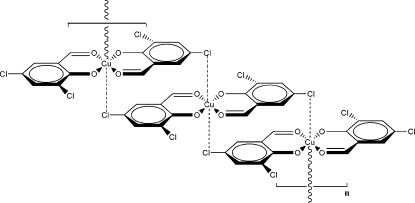


## Experimental

### 

#### Crystal data

[Cu(C_7_H_3_Cl_2_O_2_)_2_]
*M* *_r_* = 443.53
Orthorhombic, 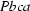

*a* = 8.1564 (8) Å
*b* = 12.4746 (12) Å
*c* = 14.7296 (14) Å
*V* = 1498.7 (3) Å^3^
*Z* = 4
Mo *K*α radiation
μ = 2.19 mm^−1^
*T* = 291 (2) K
0.14 × 0.12 × 0.10 mm

#### Data collection

Bruker SMART CCD diffractometer
Absorption correction: multi-scan (*SADABS*; Bruker, 2000 ▶) *T*_min_ = 0.750, *T*_max_ = 0.811
7424 measured reflections
1471 independent reflections
1215 reflections with *I* > 2σ(*I*)
*R*_int_ = 0.043

#### Refinement

*R*[*F* ^2^ > 2σ(*F* ^2^)] = 0.033
*wR*(*F* ^2^) = 0.103
*S* = 1.06
1471 reflections
106 parameters
H-atom parameters constrained
Δρ_max_ = 0.41 e Å^−3^
Δρ_min_ = −0.83 e Å^−3^

### 

Data collection: *SMART* (Bruker, 2000 ▶); cell refinement: *SAINT* (Bruker, 2000 ▶); data reduction: *SAINT*; program(s) used to solve structure: *SHELXTL* (Sheldrick, 2008 ▶); program(s) used to refine structure: *SHELXTL*; molecular graphics: *SHELXTL*; software used to prepare material for publication: *SHELXTL*.

## Supplementary Material

Crystal structure: contains datablocks global, I. DOI: 10.1107/S1600536808020424/bi2288sup1.cif

Structure factors: contains datablocks I. DOI: 10.1107/S1600536808020424/bi2288Isup2.hkl

Additional supplementary materials:  crystallographic information; 3D view; checkCIF report

## Figures and Tables

**Table:** 

Cu1—O1	1.906 (2)
Cu1—O2	1.943 (2)
Cu1—Cl2^ii^	3.207 (1)

**Table:** 

O1—Cu1—O1^i^	180
O1—Cu1—O2^i^	87.13 (8)
O1—Cu1—O2	92.87 (8)
O2^i^—Cu1—O2	180

Symmetry codes: (i) 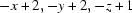; (ii) 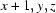; (iii) 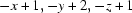.

**Fig. 1.**
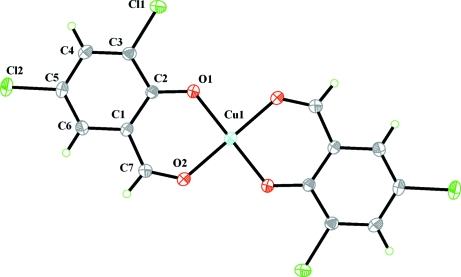
Molecular structure showing displacement ellipsoids at the 30% probability level for non-H atoms. Cu1 lies on a centre of inversion: unlabelled atoms are related to labelled atoms by the symmetry code 2-x, 2-y, 1-z.

**Fig. 2.**
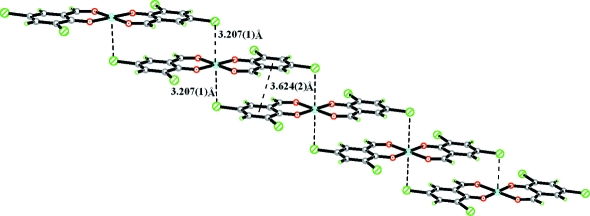
A perspective view of one chain motif, showing long Cu—Cl coordinative bonds and π–π stacking interactions (dashed lines).

## supplementary crystallographic information

### Comment

The design and synthesis of derivatives of salicylaldehyde and their metal complexes are fascinating areas of research, which can be used in a variety of studies such as drug design, life science, catalysis, and so on (Harkat *et al.*, 2008; Duan *et al.*, 2007). In our previous studies, we have reported the X-ray single-crystal structures of 3,5-dichloro-2-hydroxybenzaldehyde and 3,5-dibromo-2-hydroxybenzaldehyde (Fan *et al.*, 2008*a*, *b*). In this paper, we report the X-ray single-crystal structure of the title Cu^II^ complex.

In the title compound (Fig. 1), the coordination geometry of the central Cu^II^ ion can be described as [4 + 2]. Four Cu—O bonds from two 3,5-dichloro-2-hydroxybenzaldehyde anions constitute a distorted square coordination plane with the bond lengths varying from 1.906 (2) to 1.943 (2) Å (Table 1), which are in good agreement with those found in similar Cu^II^ complexes (Sun & Gao, 2005; Zhang *et al.*, 2006). Two adjacent Cl atoms from two 3,5-dichloro-2-hydroxybenzaldehyde anions (Cl2, symmetry codes: 1 + *x*, *y*, *z* and 1 - *x*, 2 - *y*, 1 - *z*) occupy two axial positions by weak coordinative bonds with the same Cu—Cl bond length of 3.207 (1) Å (Fig. 2). In addition, these molecules are further stabilized by π-π stacking interactions with the centroid-to-centroid separation of 3.624 (2) Å, forming one-dimensional chain motifs (Fig. 2). A dihedral angle of 45.3 (1) ° is formed between the planes of molecules in neighbouring chains.

### Experimental

A solution of Cu(OAc)_2_.H_2_O (0.1 mmol, 0.020 g) in methanol (5 ml) was added to a methanol solution (20 ml) of 3,5-dichloro-2-hydroxybenzaldehyde (0.2 mmol, 0.039 g). The resulting mixture was refluxed for 2 h, cooled and evaporated slowly at room temperature in air to give dark red single crystals suitable for X-ray diffraction measurement. Analysis calculated for C_14_H_6_O_4_Cl_4_Cu: C, 37.91. H, 1.36%; found: C, 37.85; H, 1.59%.

FT—IR (KBr pellets, cm^-1^): 3058 (*m*), 1605 (*vs*), 1510 (*s*), 1438 (*s*), 1420 (*s*), 1337 (*s*), 1217 (*s*), 1162 (*s*), 887 (*m*), 766 (*s*), 720 (*m*), 600 (*m*) and 455 (*m*).

### Refinement

H atoms bonded to C atoms were placed in geometrically idealized positions (C—H = 0.93 Å) and refined as riding atoms with *U*_iso_(H) = 1.2*U*_eq_(C).

### Crystal data

**Table:**

[Cu(C_7_H_3_Cl_2_O_2_)_2_]	*F*_000_ = 876
*M**_r_* = 443.53	*D*_x_ = 1.966 Mg m^−^^3^
Orthorhombic, *P**b**c**a*	Mo *K*α radiation λ = 0.71073 Å
Hall symbol: -P 2ac 2ab	Cell parameters from 3128 reflections
*a* = 8.1564 (8) Å	θ = 2.8–27.6º
*b* = 12.4746 (12) Å	µ = 2.19 mm^−^^1^
*c* = 14.7296 (14) Å	*T* = 291 (2) K
*V* = 1498.7 (3) Å^3^	Block, red
*Z* = 4	0.14 × 0.12 × 0.10 mm

### Data collection

**Table:**

Bruker SMART CCD diffractometer	1471 independent reflections
Radiation source: fine-focus sealed tube	1215 reflections with *I* > 2σ(*I*)
Monochromator: graphite	*R*_int_ = 0.043
*T* = 291(2) K	θ_max_ = 26.0º
φ and ω scans	θ_min_ = 2.8º
Absorption correction: multi-scan(SADABS; Bruker, 2000)	*h* = −10→10
*T*_min_ = 0.750, *T*_max_ = 0.811	*k* = −15→15
7424 measured reflections	*l* = −11→18

### Refinement

**Table:**

Refinement on *F*^2^	Secondary atom site location: difference Fourier map
Least-squares matrix: full	Hydrogen site location: inferred from neighbouring sites
*R*[*F*^2^ > 2σ(*F*^2^)] = 0.033	H-atom parameters constrained
*wR*(*F*^2^) = 0.103	*w* = 1/[σ^2^(*F*_o_^2^) + (0.0631*P*)^2^ + 0.6132*P*] where *P* = (*F*_o_^2^ + 2*F*_c_^2^)/3
*S* = 1.06	(Δ/σ)_max_ < 0.001
1471 reflections	Δρ_max_ = 0.41 e Å^−^^3^
106 parameters	Δρ_min_ = −0.83 e Å^−^^3^
Primary atom site location: structure-invariant direct methods	Extinction correction: none

### Special details

**Table:**

Geometry. All e.s.d.'s (except the e.s.d. in the dihedral angle between two l.s. planes) are estimated using the full covariance matrix. The cell e.s.d.'s are taken into account individually in the estimation of e.s.d.'s in distances, angles and torsion angles; correlations between e.s.d.'s in cell parameters are only used when they are defined by crystal symmetry. An approximate (isotropic) treatment of cell e.s.d.'s is used for estimating e.s.d.'s involving l.s. planes.
Refinement. Refinement of *F*^2^ against ALL reflections. The weighted *R*-factor *wR* and goodness of fit *S* are based on *F*^2^, conventional *R*-factors *R* are based on *F*, with *F* set to zero for negative *F*^2^. The threshold expression of *F*^2^ > σ(*F*^2^) is used only for calculating *R*-factors(gt) *etc*. and is not relevant to the choice of reflections for refinement. *R*-factors based on *F*^2^ are statistically about twice as large as those based on *F*, and *R*- factors based on ALL data will be even larger.

### Fractional atomic coordinates and isotropic or equivalent isotropic displacement parameters (Å^2^)

**Table:**

	*x*	*y*	*z*	*U*_iso_*/*U*_eq_	
Cu1	1.0000	1.0000	0.5000	0.0350 (2)	
C1	0.6397 (3)	0.9163 (2)	0.43110 (19)	0.0300 (6)	
C2	0.6929 (3)	1.0203 (2)	0.4048 (2)	0.0283 (6)	
C3	0.5807 (4)	1.0793 (2)	0.3514 (2)	0.0310 (6)	
C4	0.4281 (4)	1.0410 (2)	0.3285 (2)	0.0348 (7)	
H4	0.3569	1.0829	0.2943	0.042*	
C5	0.3807 (3)	0.9390 (2)	0.3569 (2)	0.0334 (7)	
C6	0.4844 (3)	0.8770 (2)	0.4061 (2)	0.0324 (7)	
H6	0.4528	0.8083	0.4233	0.039*	
C7	0.7396 (4)	0.8478 (3)	0.4859 (2)	0.0371 (7)	
H7	0.6969	0.7805	0.4993	0.045*	
Cl1	0.64045 (10)	1.20455 (6)	0.31242 (6)	0.0445 (3)	
Cl2	0.18463 (10)	0.89331 (8)	0.33032 (6)	0.0473 (3)	
O1	0.8330 (2)	1.06231 (16)	0.42655 (15)	0.0367 (5)	
O2	0.8767 (3)	0.86838 (18)	0.51754 (15)	0.0413 (5)	

### Atomic displacement parameters (Å^2^)

**Table:**

	*U*^11^	*U*^22^	*U*^33^	*U*^12^	*U*^13^	*U*^23^
Cu1	0.0256 (3)	0.0313 (3)	0.0481 (4)	0.0010 (2)	−0.0058 (2)	0.0057 (2)
C1	0.0251 (14)	0.0316 (15)	0.0332 (16)	0.0020 (11)	−0.0008 (12)	0.0006 (12)
C2	0.0244 (14)	0.0287 (14)	0.0318 (15)	0.0025 (11)	0.0021 (12)	0.0003 (11)
C3	0.0304 (15)	0.0287 (14)	0.0339 (15)	0.0033 (12)	0.0019 (13)	0.0006 (12)
C4	0.0309 (15)	0.0380 (16)	0.0356 (17)	0.0081 (14)	−0.0035 (13)	−0.0012 (13)
C5	0.0241 (13)	0.0436 (17)	0.0325 (15)	−0.0012 (13)	−0.0007 (12)	−0.0059 (13)
C6	0.0302 (15)	0.0319 (16)	0.0352 (16)	−0.0033 (12)	0.0001 (12)	−0.0019 (12)
C7	0.0322 (16)	0.0310 (15)	0.0481 (18)	−0.0002 (14)	−0.0035 (14)	0.0044 (13)
Cl1	0.0438 (5)	0.0313 (4)	0.0583 (5)	0.0029 (3)	−0.0019 (4)	0.0106 (3)
Cl2	0.0299 (4)	0.0576 (5)	0.0544 (5)	−0.0064 (4)	−0.0109 (3)	−0.0036 (4)
O1	0.0258 (10)	0.0321 (11)	0.0522 (13)	−0.0022 (9)	−0.0064 (9)	0.0076 (9)
O2	0.0302 (12)	0.0345 (12)	0.0592 (14)	−0.0006 (9)	−0.0110 (10)	0.0104 (10)

### Geometric parameters (Å, °)

**Table:**

Cu1—O1	1.906 (2)	C2—C3	1.413 (4)
Cu1—O1^i^	1.906 (2)	C3—C4	1.375 (4)
Cu1—O2^i^	1.943 (2)	C3—Cl1	1.735 (3)
Cu1—O2	1.943 (2)	C4—C5	1.394 (4)
Cu1—Cl2^ii^	3.207 (1)	C4—H4	0.930
Cu1—Cl2^iii^	3.207 (1)	C5—C6	1.356 (4)
C1—C6	1.408 (4)	C5—Cl2	1.742 (3)
C1—C2	1.422 (4)	C6—H6	0.930
C1—C7	1.431 (4)	C7—O2	1.238 (4)
C2—O1	1.297 (3)	C7—H7	0.930
			
O1—Cu1—O1^i^	180	C3—C4—C5	119.7 (3)
O1—Cu1—O2^i^	87.13 (8)	C3—C4—H4	120.2
O1^i^—Cu1—O2^i^	92.87 (8)	C5—C4—H4	120.2
O1—Cu1—O2	92.87 (8)	C6—C5—C4	120.5 (3)
O1^i^—Cu1—O2	87.13 (8)	C6—C5—Cl2	120.4 (2)
O2^i^—Cu1—O2	180	C4—C5—Cl2	119.1 (2)
C6—C1—C2	121.4 (3)	C5—C6—C1	120.2 (3)
C6—C1—C7	116.9 (3)	C5—C6—H6	119.9
C2—C1—C7	121.7 (3)	C1—C6—H6	119.9
O1—C2—C3	119.8 (2)	O2—C7—C1	127.0 (3)
O1—C2—C1	124.7 (3)	O2—C7—H7	116.5
C3—C2—C1	115.4 (2)	C1—C7—H7	116.5
C4—C3—C2	122.8 (3)	C2—O1—Cu1	127.25 (18)
C4—C3—Cl1	119.1 (2)	C7—O2—Cu1	126.4 (2)
C2—C3—Cl1	118.1 (2)		

Symmetry codes: (i) −*x*+2, −*y*+2, −*z*+1; (ii) *x*+1, *y*, *z*; (iii) −*x*+1, −*y*+2, −*z*+1.
